# Use of Artificial Intelligence in Vesicoureteral Reflux Disease: A Comparative Study of Guideline Compliance

**DOI:** 10.3390/jcm14072378

**Published:** 2025-03-30

**Authors:** Mehmet Sarikaya, Fatma Ozcan Siki, Ilhan Ciftci

**Affiliations:** Department of Pediatric Surgery, Faculty of Medicine, Selcuk University, Konya 42100, Turkey; doktorozcan@hotmail.com (F.O.S.); driciftci@hotmail.com (I.C.)

**Keywords:** artificial intelligence applications, vesicoureteral reflux, clinical guidelines compliance, paediatric urology, decision support systems

## Abstract

**Objective:** This study aimed to evaluate the compliance of four different artificial intelligence applications (ChatGPT-4.0, Bing AI, Google Bard, and Perplexity) with the American Urological Association (AUA) vesicoureteral reflux (VUR) management guidelines. **Materials and Methods:** Fifty-one questions derived from the AUA guidelines were asked of each AI application. Two experienced paediatric surgeons independently scored the responses using a five-point Likert scale. Inter-rater agreement was analysed using the intraclass correlation coefficient (ICC). **Results:** ChatGPT-4.0, Bing AI, Google Bard, and Perplexity received mean scores of 4.91, 4.85, 4.75 and 4.70 respectively. There was no statistically significant difference between the accuracy of the AI applications (*p* = 0.223). The inter-rater ICC values were above 0.9 for all platforms, indicating a high level of consistency in scoring. **Conclusions:** The evaluated AI applications agreed highly with the AUA VUR management guidelines. These results suggest that AI applications may be a potential tool for providing guideline-based recommendations in paediatric urology.

## 1. Introduction

The rapid advancement of artificial intelligence (AI) technologies is leading to their further integration into medical fields, providing innovative solutions for clinical decision-making and patient education [1,2,3]. The capacity of AI applications, notably chatbots, to furnish detailed and evidence-based responses is highly sophisticated [4]. In particular, platforms such as ChatGPT, Bing AI, Google Bard, and Perplexity are extensively utilized by both physicians and patients to access medical information [5].

Vesicoureteral reflux (VUR), a prevalent condition within the domain of paediatric urology, poses significant diagnostic and therapeutic challenges that necessitate adherence to established clinical guidelines. The American Urological Association (AUA) guideline on the management of primary VUR in children was last updated in 2010 and remains the most recent official guideline on this topic [6]. VUR is characterized by the retrograde flow of urine from the bladder into the ureters and kidneys. This condition, if left untreated, can lead to severe complications, including urinary tract infections, kidney scarring, hypertension, and even chronic kidney disease in severe cases [6,7]. Early diagnosis and adherence to guideline-based management are imperative to minimize the long-term morbidity associated with VUR.

The potential for AI applications to align with evidence-based guidelines has become a critical area of interest, particularly in cases such as VUR where standardized management strategies are imperative to optimize patient outcomes [8]. While these AI tools are increasingly relied upon for rapid access to medical information, questions remain about their accuracy, reliability, and consistency in adhering to established guidelines such as those provided by the AUA.

The present study aims to assess the compliance of responses generated by four widely used AI applications—ChatGPT, Bing AI, Google Bard, and Perplexity—with AUA guidelines for VUR management. The present study explores the ability of these platforms to provide guideline-compliant advice by focusing on key clinical aspects such as diagnosis, imaging recommendations, risk stratification, and treatment options.

## 2. Materials and Methods

### 2.1. Study Design

The present study was conducted with the objective of evaluating the relevance of the responses provided by four AI applications (ChatGPT 4.0, Bing AI, Google Bard, and Perplexity) to the AUA guidelines for the management of VUR in children. A total of 51 questions were developed based on the key recommendations, standards, options, and suggestions outlined in the AUA guidelines.

**Figure 1:**
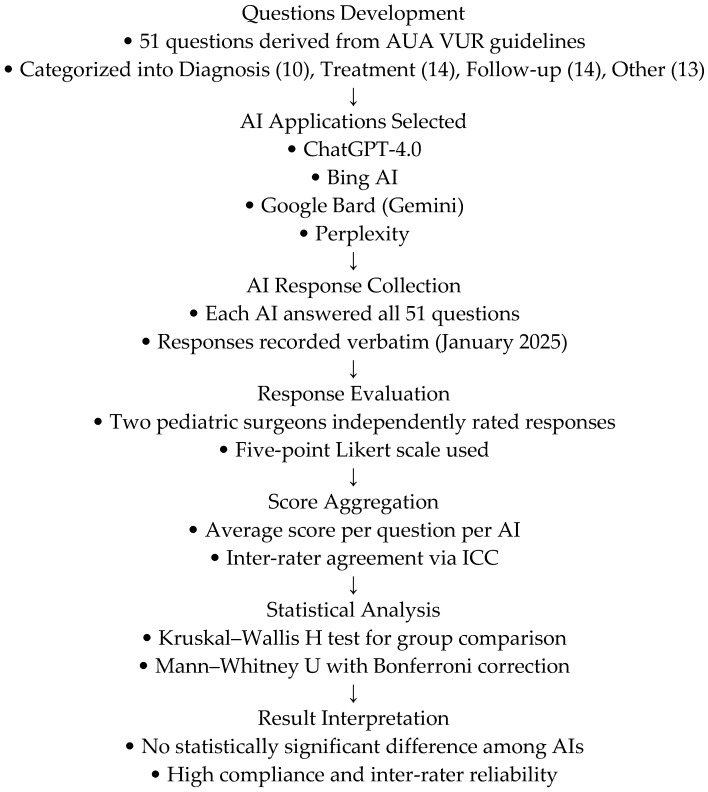
Flowchart of the process.

All AI responses were collected and evaluated in January 2025 to ensure consistency. Since AI models are continuously updated, the results of this study reflect the outputs generated during this specific period. The overall methodology of the study, including questions preparation, AI evaluation, and statistical analysis, is summarized in Figure 1**.**

### 2.2. Question Development

The questions were systematically derived from the AUA guidelines. Each recommendation from the guidelines was transformed into a corresponding question. For instance:

Guideline Recommendation: “Serum creatinine level be checked in the first-onset vesicoureteral reflux disease of a child with vesicoureteral reflux disease.”

Corresponding Question: “Should serum creatinine level be checked in the first-onset vesicoureteral reflux disease of a child with vesicoureteral reflux disease?”

These questions were methodically classified into three primary domains to ensure comprehensive coverage of the guideline’s recommendations. The distribution of questions was as follows:

Diagnosis-related inquiries: 10 questions (19.6%) addressed the diagnostic evaluation of VUR, encompassing initial assessments such as imaging, laboratory tests, and clinical evaluations.

Treatment-related questions: 14 (27.5%) of the questions addressed treatment strategies, including antibiotic prophylaxis, surgical interventions, and management of comorbidities.

Follow-up-related questions: 14 (27.5%) of the questions pertained to the follow-up care and monitoring of patients, including imaging intervals, growth assessments, and recurrence prevention.

Additional topics addressed included 13 questions (25.5%) which addressed ancillary subjects, including patient education, long-term outcomes, and familial implications of VUR.

This categorization ensured a balanced evaluation of the AI responses across different aspects of VUR management.

The study incorporated the following illustrative inquiries:

“Should renal ultrasonography be performed at the first onset of VUR in children?”

“Should height and weight be measured at the initial visit of a child with VUR?”

“Is there a difference in the risk of febrile urinary tract infections between uncircumcised and circumcised male infants with VUR?”

### 2.3. Evaluation of AI Responses

A complete list of the 51 questions derived from the AUA guidelines and used in the AI evaluation process is provided in Supplementary Table S1.

Each AI referral was sent 51 questions, and responses were recorded verbatim. Two experienced pediatric surgeons independently evaluated the responses using a 5-point Likert scale:

1—Incorrect: The AI application does not meet the criteria;

2—Inadequate: The AI application’s response is significantly inadequate in terms of the criteria;

3—Adequate: The AI application’s response is not a direct recommendation, but contains clear and accurate information;

4—Accurate: The AI application’s response is similar to the AUA guideline recommendation, but no precise recommendation is made;

5—Very accurate: The AI application’s response is directly in line with the AUA guideline recommendation.

The assessments were based on the clarity and completeness of the responses and compliance with AUA guidelines.

### 2.4. Statistical Analysis

R, version 4.2.1 (www.r-project.org, accessed on 25 January 2025) was used to perform the statistical analysis. The scores assigned to each response were presented as mean ± standard deviation (SD). Shapiro–Wilk’s test was used to assess the normality of the data. Since the data did not follow a normal distribution, the Kruskal–Wallis H test was applied to compare AI response accuracy across platforms.

To evaluate inter-rater reliability, intraclass correlation coefficients (ICCs) with 95% confidence intervals (CIs) were calculated, showing high agreement between the two independent evaluators.

Additionally, to explore potential differences between individual AI platforms, the mean of the two raters’ scores for each question was calculated and subjected to pairwise comparison using the Mann–Whitney U test with Bonferroni correction.

### 2.5. Use of Artificial Intelligence

During the preparation of this work the authors used ChatGpt-4.0 in order to process data, shape the discussion design, and place the sentences and paragraphs used in the appropriate areas. After using this tool/service, the author(s) reviewed and edited the content as needed and take(s) full responsibility for the content of the publication.

## 3. Results

A total of 51 questions about vesicoureteral reflux disease were introduced to the four AI applications (ChatGPT-4.0, Bing AI, Google Bard, and Perplexity). The mean scores of each AI application are summarized in Table 1.

The ICC between the experts were 0.975 [95% CI, 0.956–0.985] for ChatGPT, 0.915 [95% CI, 0.851–0.951] for Bing, 0.987 [95% CI, 0.976–0.992] for Gemini, and 0.976 [95% CI, 0.959–0.987] for Perplexity, demonstrating good-to-excellent agreement in evaluating the responses.

**Figure 2:**
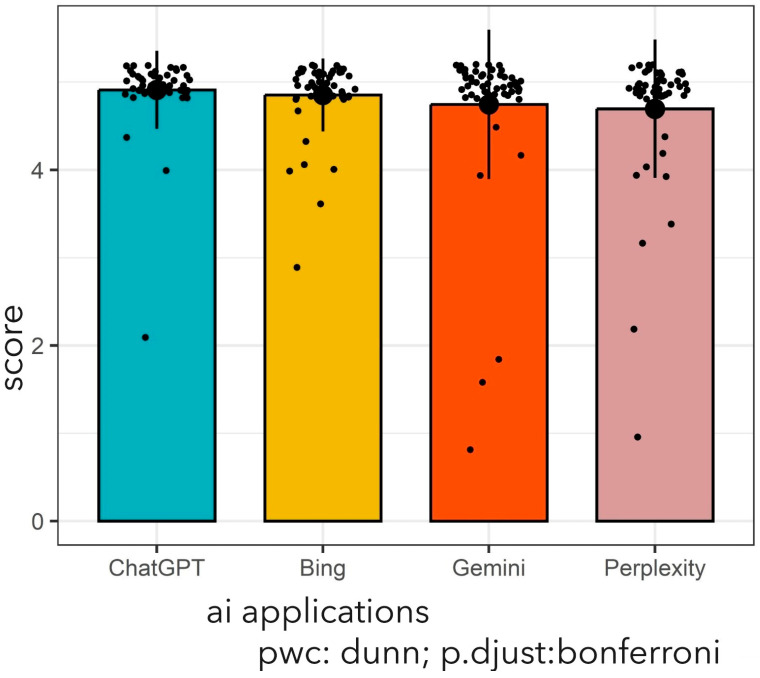
Comparison of the four AI applications regarding performance in VUR case analysis. Data are presented as mean and standard deviation. *p*-value obtained by Kruskal–Wallis H test. Dots indicate individual scores in each AI application. Kruskal–Wallis χ^2^ = 4.39, *p*-value = 0.22, *n* = 204. pwc: pairwise comparison, AI: artificial intelligence.

The average (SD) predictive score was 4.91 (0.44) for ChatGPT-4.0, 4.85 (0.42) for Bing, 4.75 (0.85) for Gemini, and 4.70 (0.79) for the Perplexity model in the management of vesicoureteral reflux disease. However, there was no statistically significant difference in the accuracy of the AI scores (Kruskal–Wallis *χ*^2^ = 4.39, *p*-value = 0.223). Figure 2 provides a detailed presentation of the average scores of four different AI applications.

Despite the absence of a statistically significant discrepancy in response accuracy among the AI applications (*p* = 0.223), this observation indicates that all four platforms demonstrate comparable efficacy in adhering to AUA guidelines for VUR management. This finding underscores their potential clinical utility.

We evaluated the scores according to the context of questions among the AI applications. However, we did not observe any statistically significant differences in the diagnosis-related responses, (*p* = 0.820) the treatment-related responses (*p* = 0.087), the follow-up responses (*p* = 0.366), and the other (*p* = 0.392) categories of response among AI applications.

**Figure 3:**
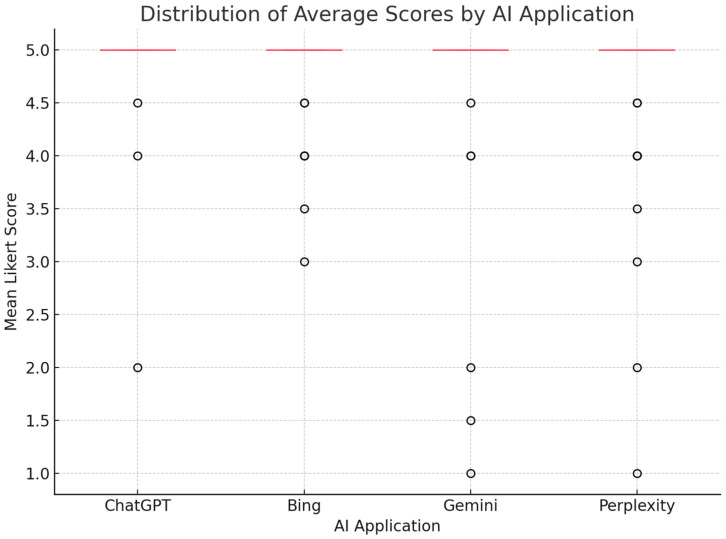
Distribution of average Likert scores for each AI application. Boxplot displays median values, interquartile ranges, and overall score variability based on 51 questions. Each score represents the mean of two independent raters. While ChatGPT demonstrated a marginally higher median score, no statistically significant difference was observed among AI applications (Kruskal–Wallis *p* = 0.2227).

A boxplot was also generated to visually demonstrate the distribution of average scores across AI applications (Figure 3). Although ChatGPT exhibited a slightly higher median score compared to other platforms, no statistically significant differences were observed, consistent with the results of the Kruskal–Wallis and post-hoc pairwise analyses.

## 4. Discussion

This study presents a comparative evaluation of four AI applications (ChatGPT, Bing AI, Google Bard, and Perplexity) in adherence to the AUA guidelines for the management of VUR. Among the platforms evaluated, ChatGPT and Perplexity demonstrated the highest compliance, with mean scores above 4.8 on a five-point Likert scale. These results highlight the potential of AI applications as valuable tools for providing guideline-based recommendations in paediatric urology. Furthermore, the high inter-rater reliability, with ICC values above 0.9 for all platforms, underlines the robustness of the scoring process and the consistency of the expert raters.

This study contributes to a growing body of research examining the application of AI, particularly large language models such as ChatGPT, in medical domains. Similar studies have evaluated the performance of ChatGPT in urological conditions, such as urolithiasis, and reported a high accuracy rate of 94.6% for responses in line with European Association of Urology (EAU) guidelines [9]. These findings are consistent with our own observations in the context of paediatric VUR, where ChatGPT demonstrated a comparable degree of agreement with the AUA guidelines.

The findings of this study are consistent with and build upon earlier research examining the performance of AI applications in the medical field. In a manner consistent with studies that have evaluated ChatGPT’s adherence to EAU guidelines for the management of urolithiasis, the present study demonstrates that AI applications, most notably ChatGPT and Perplexity, can reliably provide accurate and guideline-compliant recommendations. For instance, Cil and Dogan reported an accuracy rate of 80% for binary questions and 93.3% for descriptive questions on kidney stones, emphasising the ability of ChatGPT in urological diagnoses [10]. The adaptability of AI tools, as demonstrated by their capacity to enhance responses over time, further substantiates their integration into clinical decision-making processes, as emphasised by Cil and Doğan [10].

Moreover, the findings of this study are in alignment with those of Altintas et al., who reported the superior compliance with clinical guidelines of ChatGPT and Perplexity in comparison to other platforms [11]. These parallels underscore the mounting reliability of AI in evidence-based medicine, while underscoring the necessity for continuous professional supervision in its applications.

In the field of paediatric urology, Caglar et al. conducted a study to evaluate the capacity of ChatGPT to respond to frequently asked questions [12]. The findings revealed that 92% of the responses were entirely accurate, which is noteworthy when considering the 93.6% accuracy rate when compared to the EAU guidelines. This level of accuracy underscores the potential of AI applications as educational tools in paediatric urology and supports their integration into patient communication strategies.

Studies in other medical fields have revealed analogous trends. In a study by Önder et al., ChatGPT demonstrated an 83.3% agreement with established guidelines in the diagnosis of hypothyroidism during pregnancy, while concurrently providing comprehensive responses to inquiries regarding laboratory test interpretation [13]. Nevertheless, the readability and accessibility of these responses for the general public was often found to be inadequate in the existing studies. Consequently, there is a pressing need for concerted efforts to simplify AI-generated responses without compromising on accuracy, if the adoption of AI is to be expanded.

Whilst the majority of research has highlighted the strengths of ChatGPT, inconsistencies have been observed between AI recommendations and clinical decision-making [14,15]. In a study on bariatric surgery, ChatGPT’s recommendations met expert consensus in only 34.16% of cases, emphasising the necessity for professional supervision when employing AI tools in complex clinical decision-making [16].

The findings of the present study offer unique insights into the paediatric applications of AI, an area of research that has hitherto been the subject of only limited investigation. The demonstration of high compliance with clinical guidelines and strong inter-rater agreement among paediatric urologists serves to reinforce the potential role of AI as a reliable aid in clinical decision-making.

Whilst earlier research has chiefly concentrated on adult urological conditions, such as prostate and kidney cancer [17,18,19,20], other studies have also explored AI applications in bladder cancer [21], and urolithiasis [22]. However, research on paediatric urological conditions, particularly VUR, remains limited [23]. This study provides distinctive insights into the potential of AI in the management of paediatric VUR.

The findings of this study highlight several important implications for clinical practice, policy making and future research in paediatric urology. Primarily, the high degree of compatibility exhibited by ChatGPT and analogous AI applications with established clinical guidelines underscores their potential as supplementary resources in clinical decision-making. The integration of AI applications into routine paediatric urology practice has the potential to enhance the accessibility and precision of evidence-based recommendations, particularly in settings with limited resources where the availability of expert expertise may be limited.

Secondly, the observed limitations in readability highlight the need for dedicated AI applications that generate user-friendly responses, especially for non-medical audiences such as parents and carers.

Looking ahead, the integration of AI into paediatric urology holds substantial promise. As large language models continue to evolve, their ability to synthesize clinical guidelines and generate personalized recommendations may help streamline clinical workflows and reduce variability in decision-making [14,15]. Moreover, AI-powered tools may offer real-time support within electronic health record systems, potentially assisting physicians during patient consultations and follow-up planning [23]. In paediatric practice, AI may also contribute to individualized treatment approaches, as previously demonstrated in paediatric oncology [18]. These future applications could also include AI-driven education platforms for families, improving understanding and adherence to care strategies. Continued evaluation and ethical oversight will be essential to ensure that AI tools in paediatric urology remain accurate, transparent, and aligned with clinical standards [14].

### Limitations

This study provides significant insights into the compliance of AI applications with AUA guidelines; however, it is important to acknowledge several limitations. Firstly, the study was confined to four AI applications, which may not fully represent the extensive range of AI tools available for clinical use. Secondly, the evaluation focused exclusively on a specific area of paediatric urology and the condition of VUR, and the findings may not be generalisable to other urological or medical conditions. Thirdly, although the inter-rater reliability is high, the subjective nature of Likert scale scoring may introduce subtle biases, despite efforts to minimise them. Finally, as AI applications are constantly being updated, the responses assessed in this study may not reflect future versions of these tools, potentially limiting the long-term applicability of this study.

Future research should concentrate on expanding the scope of AI applications in paediatric urology. Research examining other paediatric conditions or comparing the performance of different AI models may provide valuable insights into optimising AI integration in clinical settings.

## 5. Conclusions

In conclusion, the present study demonstrates that AI applications, particularly ChatGPT, exhibit a high degree of compliance with the AUA guidelines for the management of paediatric VUR. The strong inter-rater consensus among expert raters emphasises the reliability of these tools as aids in clinical practice. While AI applications hold great promise in improving adherence to guidelines and facilitating evidence-based decision-making, efforts to increase the readability and contextualisation of their outputs are crucial to maximise their usability. The integration of AI technology into paediatric urology practice holds significant potential for enhancing patient care and education, paving the way for more equitable and efficient healthcare delivery.

## Figures and Tables

**Table 1:** Scores of each AI application.

	ChatGPT	Bing	Gemini	Perplexity	*p*-Value
General (Total) (*n* = 51)	4.91 ± 0.44	4.85 ± 0.42	4.75 ± 0.85	4.70 ± 0.79	0.223
Diagnosis (*n* = 10)	4.95 ± 0.16	4.80 ± 0.48	4.95 ± 0.16	4.55 ± 1.26	0.820
Follow-up (*n* = 14)	5.00 ± 0.00	4.75 ± 0.58	5.00 ± 0.00	4.71 ± 0.64	0.087
Treatment (*n* = 14)	4.71 ± 0.83	4.86 ± 0.36	4.11 ± 1.47	4.57 ± 0.85	0.366
Other (*n* = 13)	5.00 ± 0.00	5.00 ± 0.00	5.00 ± 0.00	4.92 ± 0.28	0.392

*p*-value obtained by Kruskal–Wallis test; *n* shows number of question.

## Data Availability

Data will be made available upon reasonable request. The full set of 51 questions used in this study is available as Supplementary Table S1.

## Supplementary Materials

The following supporting information can be downloaded at https://www.mdpi.com/article/10.3390/jcm14072378/s1: Table S1: List of 51 Questions Used for AI Evaluation.
