# Supplementary material for: Use of Artificial Intelligence in Vesicoureteral Reflux Disease: A Comparative Study of Guideline Compliance
Source: J Clin Med. 2025 Mar 30;14(7):2378. doi: 10.3390/jcm14072378 (PMC11989457; doi:10.3390/jcm14072378)
Supplement: Supplementary file 1 [file jcm-14-02378-s001.zip › jcm-3549038-supplementary.pdf]

Supplementary Table S1. List of 51 Questions Used for AI Evaluation

No	Category	Question
1	Diagnosis	What is vesicoureteral reflux disease?
2	Other	Do vesicoureteral reflux disease and urinary tract infections negatively affect the general health and kidneys of affected children?
3	Diagnosis	Should height and weight be measured at the first visit of a child with vesicoureteral reflux disease?
4	Diagnosis	Should blood pressure be measured at the first onset of vesicoureteral reflux disease in a child with vesicoureteral reflux disease?
5	Other	Should a child with vesicoureteral reflux disease be evaluated for renal anomalies in the first episode of vesicoureteral reflux disease?
6	Diagnosis	Should serum creatinine level be checked in the first-onset vesicoureteral reflux disease of a child with vesicoureteral reflux disease?
7	Other	Should urinalysis be done at the first onset of vesicoureteral reflux disease in a child with vesicoureteral reflux disease?
8	Other	Should a urine culture be taken if the urinalysis of a child with vesicoureteral reflux disease shows infection at the first onset of vesicoureteral reflux disease?
9	Follow-Up	Should renal ultrasonography be performed at the first onset of vesicoureteral reflux disease in a child with vesicoureteral reflux disease?
10	Follow-Up	Should DMSA imaging be performed at the first onset of vesicoureteral reflux disease in a child with vesicoureteral reflux disease?
11	Diagnosis	What is Bladder Bowel

		Dysfunction (BBD)?
12	Diagnosis	Which symptoms occur in patients with Bladder Bowel Dysfunction?
13	Diagnosis	Should symptoms indicating bladder bowel dysfunction be sought in the initial evaluation of a child with vesicoureteral reflux disease?
14	Other	Should family and patient education about vesicoureteral reflux disease be provided?
15	Other	What are the goals of management of the child with vesicoureteral reflux disease?
16	Other	Should there be separate guidelines for the management of the child with vesicoureteral reflux disease for children younger than 1 year and older than 1 year?
17	Treatment	Should continuous antibiotic prophylaxis be recommended in children younger than 1 year with vesicoureteral reflux disease with a history of febrile urinary tract infection?
18	Treatment	Should continuous antibiotic prophylaxis be recommended for children with Grade 3-5 reflux detected in screening without a history of febrile urinary tract infection?
19	Treatment	Should continuous antibiotic prophylaxis be recommended for children with Grade 1-2 reflux detected in screening without a history of febrile urinary tract infection?
20	Follow-Up	Is there a difference in the frequency of urinary tract infection between uncircumcised male infants younger than 1 year and circumcised male infants with vesicoureteral reflux disease?
21	Follow-Up	Should circumcision be recommended for uncircumcised male infants younger than 1 year of age with vesicoureteral reflux disease?
22	Treatment	Should surgery or treatment of bladder bowel dysfunction be

		the first approach in a child with vesicoureteral reflux disease?
23	Treatment	Should antibiotic prophylaxis be given in a child with bladder bowel dysfunction (BBD) and vesicoureteral reflux disease?
24	Treatment	Should antibiotic prophylaxis be given in children older than 1 year with urinary tract infection and vesicoureteral reflux disease if there is no bladder bowel dysfunction?
25	Treatment	Should continuous antibiotic prophylaxis be given in the follow-up of children older than 1 year without bladder bowel dysfunction (BBD), recurrent urinary tract infection and renal cortical damage?
26	Treatment	Can a patient older than 1 year with bladder bowel dysfunction, recurrent urinary tract infection and renal cortical damage be kept under observation without prophylaxis or additional treatment?
27	Treatment	Is there a difference in the risk of febrile urinary tract infection between those who underwent open surgery for vesicoureteral reflux disease and those who were followed up under antibiotic prophylaxis?
28	Follow-Up	How often should blood pressure, height and weight monitoring be performed in the follow-up of a child with vesicoureteral reflux disease? (annual follow-up)
29	Follow-Up	How often should urinalysis and urine culture be performed for proteinuria and bacteriuria in a child with vesicoureteral reflux disease? (annually)
30	Follow-Up	How often should urinary tract ultrasonography be performed to observe renal enlargement and scarring in a child with

		vesicoureteral reflux disease? (annual follow-up)
31	Follow-Up	How often should Voidine cystourethrography be performed in a child with VEZICOURETERAL REFLOWER DISEASE followed up with an observational approach? (Every 12-24 months)
32	Other	Is the rate of spontaneous resolution high in children younger than 1 year of age with grade 1-2 VEZICOURETERAL REFLUSION DISEASE?
33	Other	If there is clinical evidence of bladder/bowel dysfunction, should bladder/bowel dysfunction be treated, preferably before any surgical intervention for vesicoureteral reflux disease?
34	Follow-Up	Should DMSA imaging be recommended when renal ultrasound is abnormal, when there is further concern about scarring (i.e., sudden urinary tract infection, grade III-V VEZICOURETERAL REFLOWERING DISEASE), or when serum creatinine is elevated?
35	Treatment	If a symptomatic breakthrough urinary tract infection occurs (manifested by fever, dysuria, frequent urination, failure to thrive or malnutrition), should a change in treatment be recommended?
36	Treatment	If urinary tract infection with symptomatic breakthrough occurs, should the clinical scenario guide the choice of treatment alternatives?
37	Other	If a symptomatic excretory urinary tract infection occurs, should this include the degree of vesicoureteral reflux disease, the degree of renal scarring if present, and evidence of abnormal emptying patterns (bladder/bowel dysfunction) and parental preferences that

		may contribute to the urinary tract infection?
38	Treatment	Should continuous antibiotic prophylaxis be recommended for patients who develop a febrile urinary tract infection who have not received prior prophylaxis?
39	Treatment	Are patients who undergo open surgical repair for vesicoureteral reflux disease less likely to have a febrile urinary tract infection than those who receive continuous antibiotic prophylaxis?
40	Other	Should open surgical ureteral reimplantation or endoscopic injection of bulking agents be considered for curative intervention in patients with vesicoureteral reflux disease with febrile urinary tract infection?
41	Follow-Up	Following open surgical or endoscopic procedures for vesicoureteral reflux disease, should renal ultrasound be performed to assess obstruction?
42	Follow-Up	Should postoperative excretory cystography of bulking agents after endoscopic injection be recommended?
43	Diagnosis	After spontaneous or surgical resolution of vesicoureteral reflux disease, if one of the kidneys is found to be abnormal by ultrasound or DMSA scan, should general evaluation be recommended throughout adolescence, including follow-up of blood pressure, height, weight and urinalysis for protein and urinary tract infection?
44	Diagnosis	Should evaluation for bladder/bowel dysfunction or recurrent vesicoureteral reflux disease be recommended if febrile urinary tract infection occurs after resolution or surgical treatment of vesicoureteral reflux disease?

45	Other	Should the long-term concerns of hypertension (especially in pregnancy), loss of kidney function, recurrent urinary tract infection and familial vesicoureteral reflux disease in the child's siblings and children be discussed with the family and communicated to the child at an appropriate age?
46	Follow-Up	In siblings of children with vesicoureteral reflux disease, if there is ultrasound evidence of renal cortex abnormalities or renal size asymmetry, or if the untested sibling has a history of urinary tract infection, should an excretory cystoureterogram or radionuclide cystogram be recommended?
47	Follow-Up	Should an excretory cystourethrogram be recommended for children with high-grade (Fetal Urological Society grades 3 and 4) hydronephrosis, hydroureter or abnormal bladder on ultrasound (late prenatal or postnatal) or who develop urinary tract infection during observation?
48	Diagnosis	What is the incidence of vesicoureteral reflux disease in newborns with antenatal hydronephrosis (16%)?
49	Follow-Up	Should a voiding cystourethrogram be performed for children with high-grade hydronephrosis (grade 3-4), hydroureter or abnormal bladder on ultrasound or who develop urinary tract infection during observation?
50	Treatment	Is there a difference between the success rates of endoscopic injection therapy and open surgical repairs for vesicoureteral reflux disease (83%-98%)?
51	Other	Can reflux resolve spontaneously in children

		under 1 year of age (50% resolve within 2 years)?
